# Supplementary material for: The Changes of Amino-Acid Metabolism between Wheat and Rice during Early Growth under Flooding Stress
Source: Int J Mol Sci. 2024 May 11;25(10):5229. doi: 10.3390/ijms25105229 (PMC11121113; doi:10.3390/ijms25105229)
Supplement: Supplementary file 1 [file ijms-25-05229-s001.zip › Suplemental Figures.pdf]

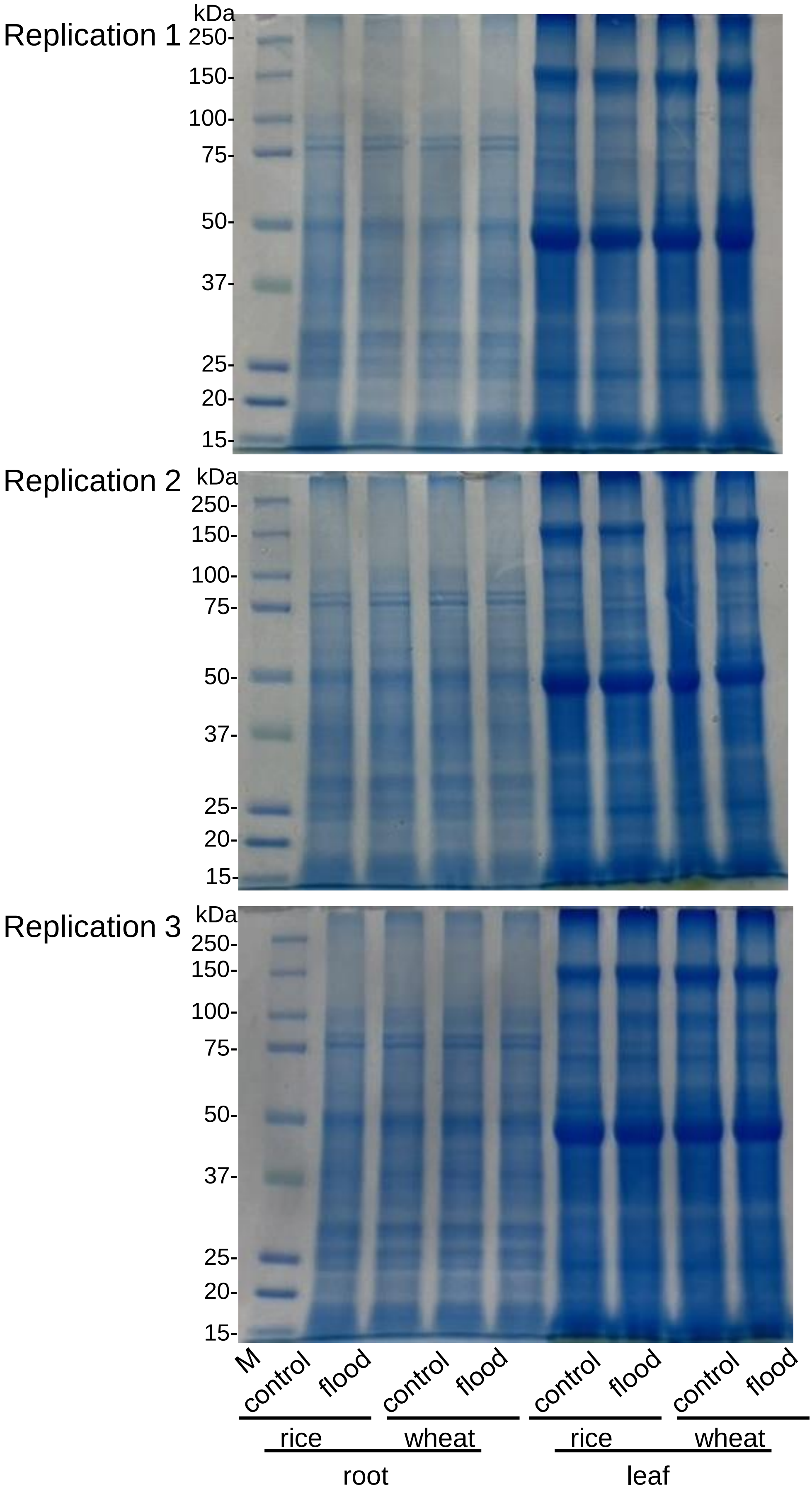

Figure S1. The Coomassie-brilliant blue staining patterns of proteins used for immunoblot analysis. Experiments were performed with biologically triplicates for each treatment. Quantified proteins (10 µg) in root and leaf of wheat treated with or flooding stress were separated by electrophoresis on a 10% SDS-polyacrylamide gel. Coomassie-brilliant blue staining was used as a loading control, which are used in Figure 5A. M means marker proteins (kDa)

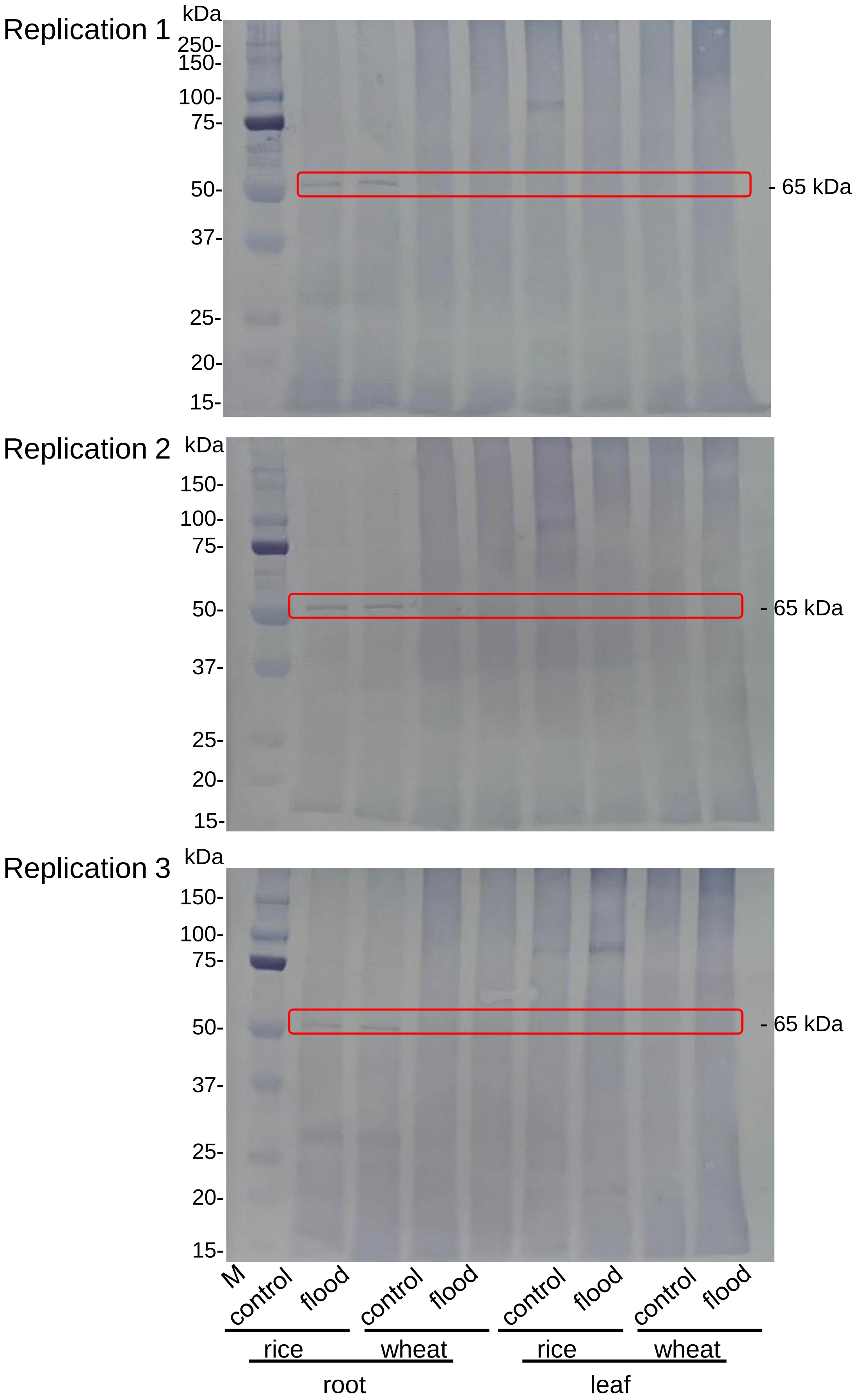

Figure S2. Blots of the entire membrane with glutamate decarboxylase antibody, which are used in Figure 5B. Experiments were performed with biologically triplicates for each treatment. “M” means marker proteins.

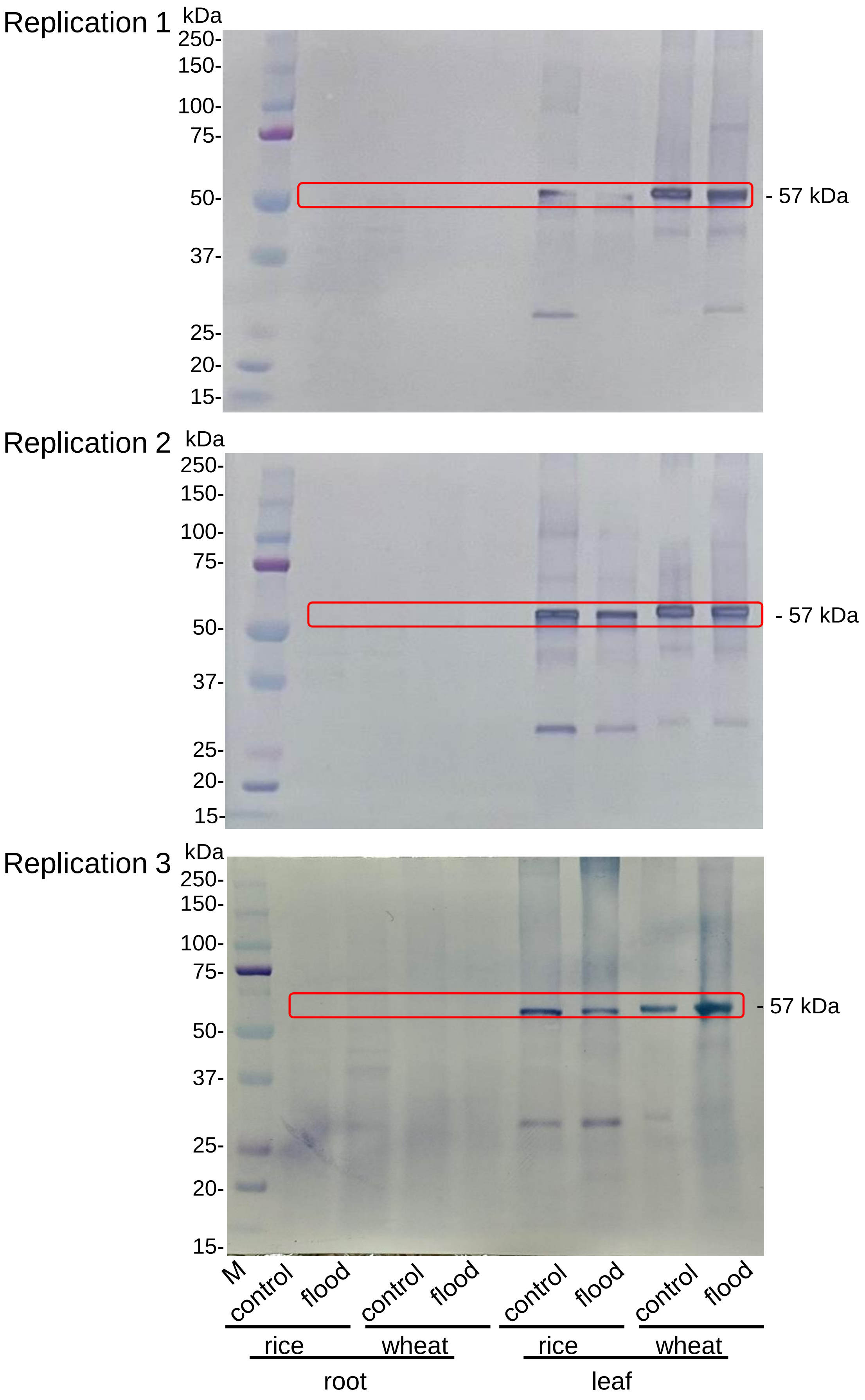

Figure S3. Blots of the entire membrane with succinyl semialdehyde dehydrogenase antibody, which are used in Figure 5C. Experiments were performed with biologically triplicates for each treatment. “M” means marker proteins.

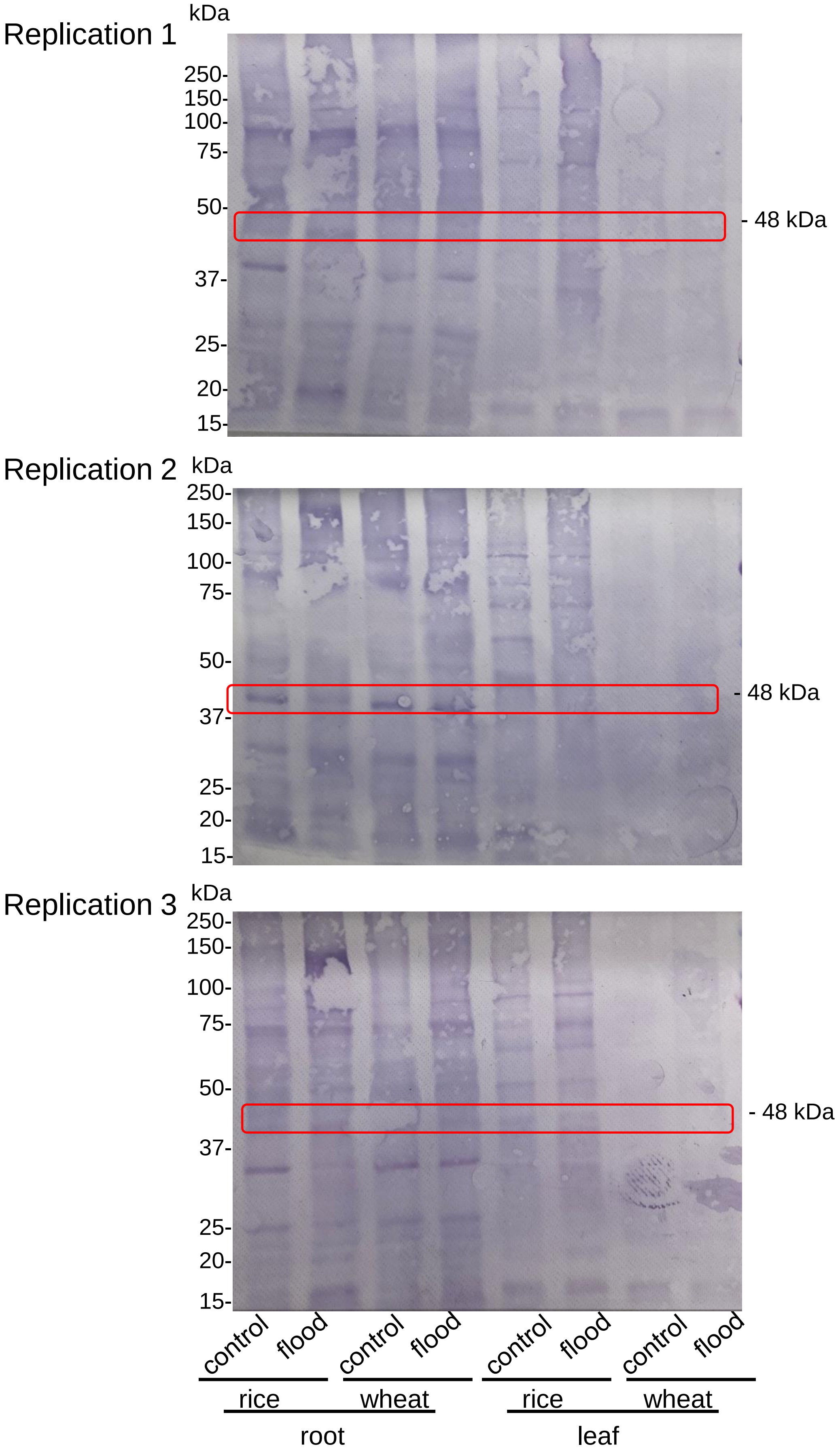

Figure S4. Blots of the entire membrane with alcohol dehydrogenase antibody, which are used in Figure 6A. Experiments were performed with biologically triplicates for each treatment.

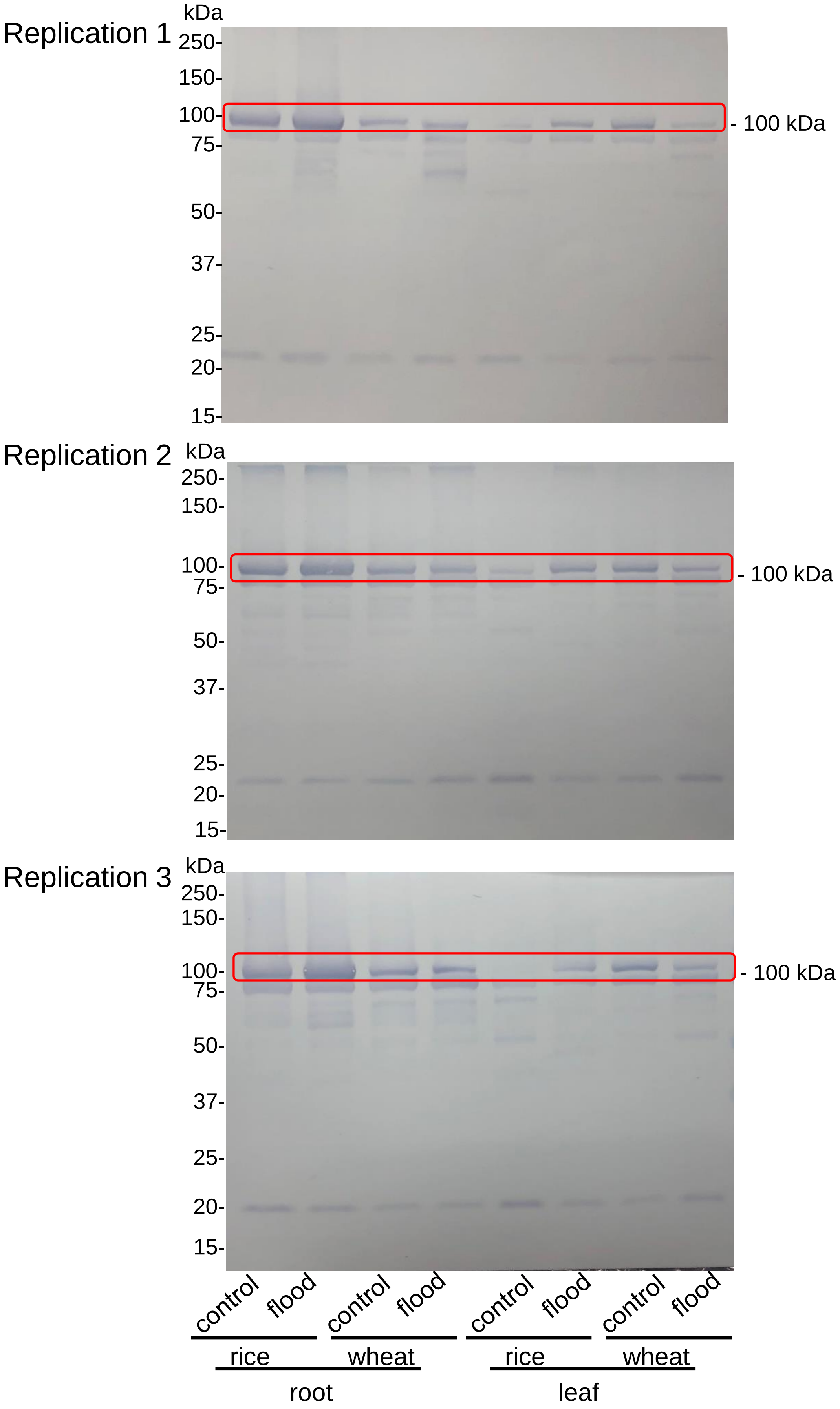

Figure S5. Blots of the entire membrane with pyruvate carboxylase antibody, which are used in Figure 6B. Experiments were performed with biologically triplicates for each treatment. “M” means marker proteins.

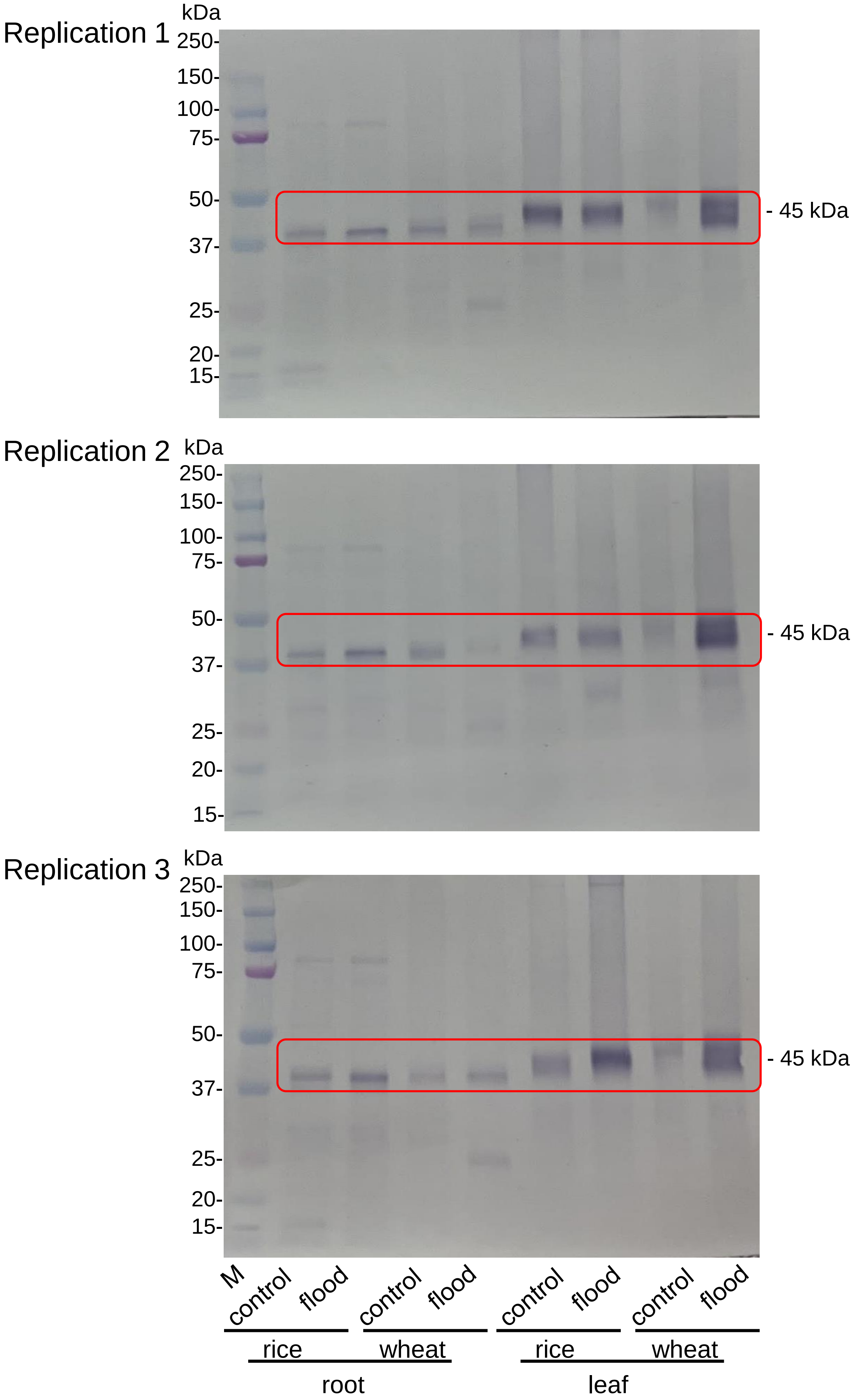

Figure S6. Blots of the entire membrane with aspartate aminotransferase antibody, which are used in Figure 6C. Experiments were performed with biologically triplicates for each treatment. “M” means marker proteins.
