# Supplementary material for: The Changes of Amino-Acid Metabolism between Wheat and Rice during Early Growth under Flooding Stress
Source: Int J Mol Sci. 2024 May 11;25(10):5229. doi: 10.3390/ijms25105229 (PMC11121113; doi:10.3390/ijms25105229)
Supplement: Supplementary file 1 [file ijms-25-05229-s001.zip › Suplemental Table.pdf]

Table S1. List of contents of amino acids in root of rice and wheat

	rice (nmol/mL)				wheat (nmol/mL)			
	control		flood		control		flood	
	mean	± SD	mean	± SD	mean	± SD	mean	± SD
P-Ser	9.52	0.49	12.96	0.18	8.12	1.02	10.91	0.50
PEA	0.00	0.00	30.15	0.71	0.00	0.00	58.59	1.85
Asp	161.19	5.50	414.45	8.35	174.75	18.94	129.00	0.63
Thr	35.89	0.39	89.49	6.87	83.44	11.76	189.28	6.34
Ser	109.30	1.43	224.05	22.21	115.59	13.59	186.88	9.71
Asn	28.31	0.00	82.19	17.45	525.75	133.52	1465.73	123.76
Glu	202.72	62.41	436.12	26.29	305.14	25.55	610.62	47.92
Gln	71.78	37.78	155.62	4.09	573.29	194.15	628.38	57.82
Gly	20.43	24.76	123.61	42.42	77.29	34.79	146.18	2.18
Ala	69.00	4.76	667.58	99.78	263.65	60.28	2816.53	197.49
a-ABA	0.00	0.00	12.02	1.87	0.00	0.00	30.27	2.13
Val	85.44	6.67	191.92	15.74	140.60	26.47	358.29	6.21
Met	91.54	4.15	97.68	1.68	93.30	3.31	105.34	2.62
Ile	74.00	8.59	137.35	12.52	128.17	22.91	281.43	6.09
Leu	85.96	11.50	184.66	22.43	164.33	31.95	384.26	6.93
Tyr	21.29	0.87	66.53	7.14	28.68	4.91	82.11	1.52
Phe	14.90	1.11	54.45	6.24	19.45	3.04	73.76	1.24
GABA	43.75	9.97	1191.41	91.33	73.66	10.75	1671.63	128.41
MEA	0.00	0.00	70.10	23.39	0.00	0.00	0.00	0.00
NH_3	343.37	135.66	507.34	62.85	387.68	69.54	316.77	34.85
Orn	0.00	0.00	17.17	5.73	0.00	0.00	0.00	0.00
1M-His	0.00	0.00	57.93	5.71	0.00	0.00	90.45	3.55
His	45.59	0.91	110.20	6.03	68.81	9.19	146.75	5.64
Lys	28.94	1.42	121.67	11.70	19.71	5.37	131.15	7.96
Arg	24.96	2.14	92.79	5.62	0.00	0.00	104.08	4.31
Pro	53.91	3.76	108.25	22.68	84.40	19.38	351.72	31.21

The data are presented as mean ± S.D. from 3 independent biological replicates.
